# A Central Bioactive Region of LTBP-2 Stimulates the Expression of TGF-β1 in Fibroblasts via Akt and p38 Signalling Pathways

**DOI:** 10.3390/ijms18102114

**Published:** 2017-10-09

**Authors:** Mohamed A. Sideek, Joshua Smith, Clementine Menz, Julian R. J. Adams, Allison J. Cowin, Mark A. Gibson

**Affiliations:** 1 Discipline of Anatomy and Pathology, School of Medicine, University of Adelaide, Adelaide, SA 5005, Australia; arshadsideek@iium.edu.my (M.A.S.); joshua.smith.phd@gmail.com (J.S.); clementine.menz@gmail.com (C.M.); julian.robert.adams@gmail.com (J.R.J.A.); 2 Department of Physical Rehabilitation Sciences, Kulliyyah of Allied Health Sciences, International Islamic University Malaysia, 25200 Kuantan, Pahang, Malaysia; 3 Regenerative Medicine, Future Industries Institute, University of South Australia, Adelaide, SA 5095, Australia; Allison.Cowin@unisa.edu.au

**Keywords:** LTBP-2, TGF-β, fibroblast, p38 MAPK, Akt, fibrosis

## Abstract

Latent transforming growth factor-β-1 binding protein-2 (LTBP-2) belongs to the LTBP-fibrillin superfamily of extracellular proteins. Unlike other LTBPs, LTBP-2 does not covalently bind transforming growth factor-β1 (TGF-β1) but appears to be implicated in the regulation of TGF-β1 bioactivity, although the mechanisms are largely unknown. In experiments originally designed to study the displacement of latent TGF-β1 complexes from matrix storage, we found that the addition of exogenous LTBP-2 to cultured human MSU-1.1 fibroblasts caused an increase in TGF-β1 levels in the medium. However, the TGF-β1 increase was due to an upregulation of TGF-β1 expression and secretion rather than a displacement of matrix-stored TGF-β1. The secreted TGF-β1 was mainly in an inactive form, and its concentration peaked around 15 h after addition of LTBP-2. Using a series of recombinant LTBP-2 fragments, the bioactivity was identified to a small region of LTBP-2 consisting of an 8-Cys motif flanked by four epidermal growth factor (EGF)-like repeats. The LTBP-2 stimulation of TGF-β expression involved the phosphorylation of both Akt and p38 mitogen-activated protein kinase (MAPK) signalling proteins, and specific inactivation of each protein individually blocked TGF-β1 increase. The search for the cell surface receptor mediating this LTBP-2 activity proved inconclusive. Inhibitory antibodies to integrins β1 and αVβ5 showed no reduction of LTBP-2 stimulation of TGF-β1. However, TGF-β1 upregulation was partially inhibited by anti-αVβ3 integrin antibodies, suggestive of a direct or indirect role for this integrin. Overall, the study indicates that LTBP-2 can directly upregulate cellular TGF-β1 expression and secretion by interaction with cells via a short central bioactive region. This may be significant in connective tissue disorders involving aberrant TGF-β1 signalling.

## 1. Introduction

Transforming growth factor-β (TGF-β) is a cytokine that exists as three isoforms (TGF-β1, TGF-β2, and TGF-β3) and regulates numerous physiological processes, including cell growth, differentiation, adhesion, apoptosis, and modulation of extracellular matrix [1,2]. TGF-β can act as a growth inhibitor in some cells, including keratinocytes and lung epithelial cells, but can also promote growth in cells such as smooth muscle cells, mesodermal cells, and fibroblasts [3,4]. When over-expressed, TGF-β exhibits pro-fibrotic effects, including excess matrix production, transdifferentiation of fibroblasts into myofibroblasts, and inhibition of apoptosis, contributing to fibrotic diseases such as pulmonary fibrosis, liver cirrhosis, and keloid scar formation [5,6]. Disturbance of TGF-β pathways is also associated with other pathological states, including tumour cell growth, cancer, and autoimmune diseases [7]. TGF-β null mice die shortly after birth due to developmental abnormalities and excessive inflammatory response in the heart and lungs [8].

TGF-β is synthesized as a small latent complex of dimers of TGF-β non-covalently bound to its pro-peptide, called the latency-associated peptide. This small complex can bind covalently to latent transforming growth factor-β-1 binding proteins (LTBPs), forming the large latent complex [9]. The large latent complex controls the storage and activation of latent TGF-β by binding to fibrillin-rich microfibrils in the extracellular matrix [10]. The LTBP/fibrillin superfamily (LTBPs 1–4 and fibrillins 1–3) have similar structures, consisting predominantly of tandem epidermal growth factor (EGF)-like 6-cysteine repeats interspersed with characteristic 8-cysteine motifs [10,11]. Unlike other LTBPs, LTBP-2 is unable to associate and bind covalently to latent TGF-β, since it lacks the required binding consensus sequence [12,13]. The function of LTBP-2 is poorly understood, although it is expressed abundantly in tissues such as lung, heart, aorta, placenta, liver, and skeletal muscle tissues [14,15]. LTBP-2 null mice were originally reported to die during embryo implantation [16]. However, a LTBP-2 null strain has recently been reported that survives to adulthood with lens luxation caused by an abnormal formation of fibrillin-rich ciliary zonules [17], which is consistent with the mild ocular phenotypes shown by LTBP-2 null humans [18,19]. *LTBP2* gene mutations have been linked to Weill–Marchesani syndrome (WMS) [20], characterized by ocular and skeletal deformities and thick fibrotic skin, containing disorganised fibrillin-1 microfibrillar networks. *FBN1* gene mutations also cause WMS [21], indicating that the disease mechanism involves both proteins. Fibrillin-1 modulation of TGF-β storage and activation is well-documented [1,22,23]. Thus, it is possible that LTBP-2 may also influence TGF-β bioavailability, supported by observations that some individuals with LTBP-2 mutations exhibit Marfan Syndrome-like characteristics linked to aberrant TGF-β upregulation [18,19]. LTBP-2 may also be involved in the regulation of latent TGF-β storage and activation in the extracellular matrix. In a rat model, the treatment of astrocytes with antisense LTBP-2 oligonucleotides resulted in a downregulation of both LTBP-2 mRNA expression and TGF-β activity, strongly suggesting that LTBP-2 may regulate TGF-β activation [24]. Hirani, Hanssen, and Gibson [14] reported that LTBP-2 specifically binds to fibrillin-1 rather than fibrillin-2 and that a C-terminal fragment of LTBP-2 blocks the interaction of LTBP-1 with fibrillin-1 in vitro [14]. These findings led to the hypothesis that LTBP-2 might indirectly regulate TGF-β bioavailability by releasing LTBP-1 from microfibrils through competitive binding for fibrillin-1.

Given this evidence, it seems clear that LTBP-2 plays some role in regulating TGF-β bioactivity, but the mechanism remains unclear. In this paper, we report that a central bioactive region of LTBP-2 can upregulate TGF-β1 expression in fibroblasts and that the mechanism involves both Akt and p38 mitogen-activated protein kinase (MAPK) signalling pathways. The cell surface receptor(s) involved in the upregulation remain(s) undefined, but may be complexed with αVβ3 integrin.

## 2. Results

### 2.1. Exogenous LTBP-2 Stimulates Expression and Secretion of TGF-β1 in MSU-1.1 Fibroblasts

**Figure 1:**
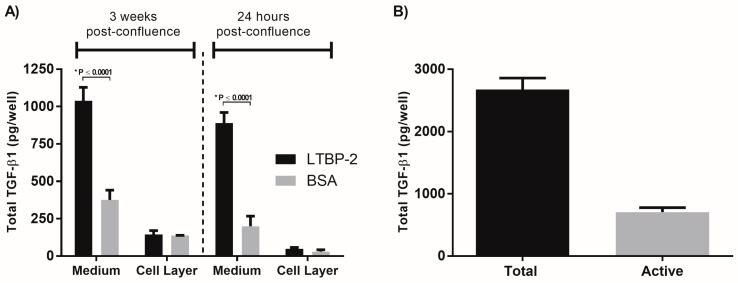
Exogenous latent transforming growth factor-β-1 binding protein-2 (LTBP-2) increases transforming growth factor-β1 (TGF-β1) in conditioned medium, which is independent of extracellular matrix. (**A**) MSU-1.1 cells were cultured for 24 h or 3 weeks post-confluence, then incubated in serum-free medium overnight containing 10 µg/mL LTBP-2 (black columns) or bovine serum albumin (BSA) control (grey columns) prior to TGF-β1 assay. Total TGF-β in the conditioned medium and cell layer was measured by ELISA (see Materials and Methods). LTBP-2 caused a significant increase in TGF-β1 without the presence of extensive extracellular matrix; (**B**) The secreted TGF-β1 is mainly in an inactive form. The medium from 3-week post-confluence LTBP-2-treated cultures was analysed with and without acid treatment for total and active TGF-β1, respectively. Approximately 70% of the TGF-β1 in the conditioned medium was inactive. Quantified data are expressed as mean ± SD (standard deviation). Statistical significance was determined by paired Student’s *t* test, * *p* < 0.05.

To study the potential role of LTBP-2 in the modulation of TGF-β matrix storage and activation, MSU-1.1 cells were selected since these cells have high levels of expression of fibrillin-1, LTBP-1, and TGF-β1 but low expression of LTBP-2, and the cells form an extensive fibrillin microfibril network [25]. The initial experiment was designed to determine if LTBP-2 could displace LTBP-1/TGF-β1 complexes from their attachment to fibrillin microfibrils. MSU-1.1 cells were cultured to 3 weeks post-confluence, to allow for the formation of an extensive extracellular matrix rich in fibrillin-1 microfibrils. After incubation with exogenous LTBP-2 for 16 h, TGF-β1 was significantly elevated in the medium, with an approximately threefold increase compared to control cells incubated with bovine serum albumin (BSA) (Figure 1A). However, the TGF-β1 content of the cell layer showed no significant difference between treated and control cultures, indicating that the increase in TGF-β1 may represent a newly synthesised protein rather than protein displaced from the microfibrils in the matrix.

**Figure 2:**
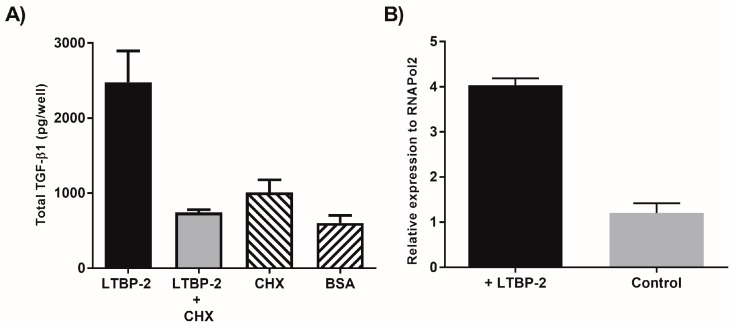
LTBP-2 upregulates TGF-β1 expression in MSU-1.1 cells. *(***A**) MSU-1.1 cells were cultured for 24 h post-confluence, then incubated for 16 h in serum-free medium with 10 µg/mL LTBP-2 or both LTBP-2 and 10 µg/mL cycloheximide (CHX). The negative controls included 10 µg/mL BSA or cycloheximide only. The conditioned medium was analysed for TGF-β1 content (see Materials and Methods); (**B**) MSU-1.1 cells were grown to 24 h post-confluence, then incubated for 16 h with 10 µg/mL full length LTBP-2 or 10 µg/mL BSA control. Total RNA was harvested from the cells, then reverse transcribed into cDNA for use in qPCR. The cDNA was analysed for TGF-β1, and normalized to RNAPolII. LTBP-2 stimulated a fourfold increase in the levels of TGF-β1 mRNA compared to the BSA control.

To test this idea, exogenous LTBP-2 was added to MSU-1.1 cells grown for only 24 h post-confluence such that only minimal fibrillin microfibrils would be present [26]. Again, TGF-β1 was highly elevated in the medium relative to control cultures, but there was little difference in cell layer between the treated and control cells (Figure 1A). An analysis of the TGF-β1 released into the medium of 3 weeks post-confluence cultures indicated that approximately 70% of the TGF-β1 was in latent (complexed) form (Figure 1B). The addition of LTBP-2 to 24 h post-confluence cells was repeated in the presence of the translation inhibitor cycloheximide [27]. Cycloheximide effectively blocked the LTBP-2-induced elevation of TGF-β1 in the medium, indicating that the increase involved new protein synthesis (Figure 2A). Quantitative PCR for TGF-β1 expression was also performed on RNA extracted from MSU-1.1 cells after 15 h of incubation with exogenous LTBP-2 or BSA control (Figure 2B). The LTBP-2-treated cells showed a fourfold elevation in TGF-β1 mRNA compared to BSA treated controls, indicating that LTBP-2 greatly stimulated TGF-β1 expression in these fibroblasts. Overall, the results indicate that LTBP-2 greatly upregulates the release of TGF-β1 into the culture medium, but the mechanism primarily involves an upregulation of *TGFB1* gene expression rather than an increased secretion of existing TGF-β1 or a displacement of existing latent TGF-β1 from fibrillin-microfibrils.

### 2.2. Time Course for LTBP-2 Stimulation of TGF-β1 Upregulation

**Figure 3:**
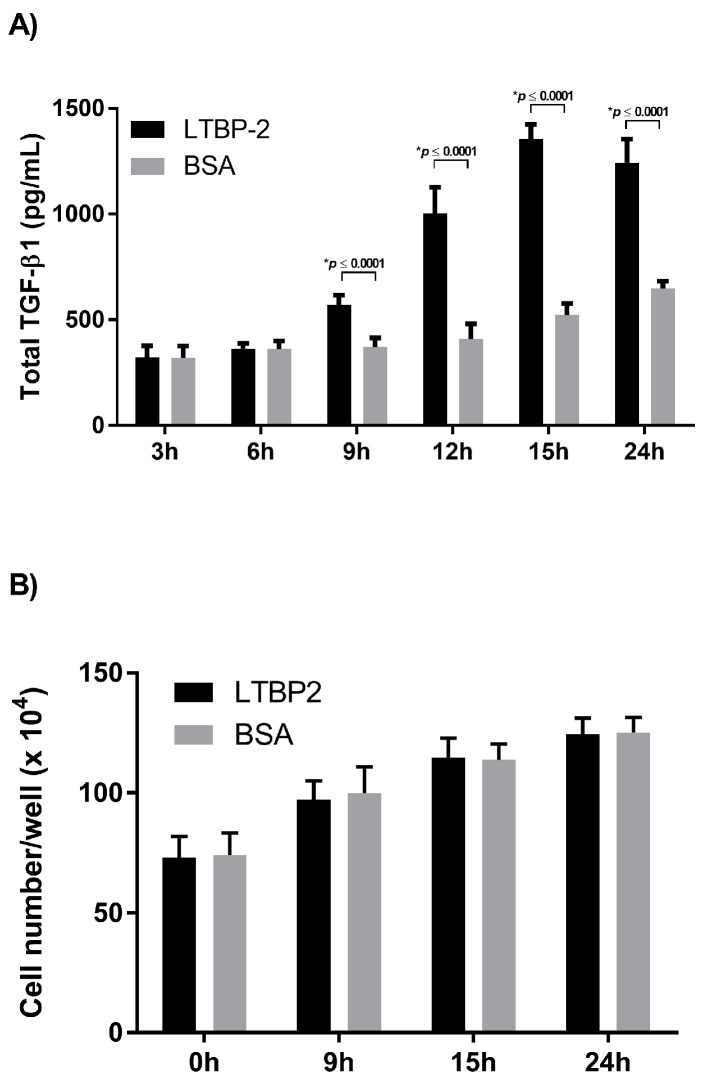
Time course for LTBP-2 stimulation of TGF-β1 production. MSU-1.1 cells were grown on 12-well plates to 24 h post-confluence and incubated with LTBP-2 (10 µg/mL) for various times (3–24 h). BSA at the same concentration was added to control wells. (**A**) TGF-β1 accumulation in the medium was measured by ELISA as described in materials and methods. The TGF-β1 concentration peaked at around 15 h. The mean values of two independent experiments are shown (±SD); (**B**) Total cell counts at selected time points. Note that LTBP-2 had no effect on cell proliferation above the BSA controls.

The optimal concentration of exogenous LTBP-2 was determined by testing a range of concentrations from 1 to 12.5 µg/mL. The level of TGF-β1 expression appeared to saturate at a LTBP-2 concentration of around 10 µg/mL, with TGF-β1 elevated around fourfold above the BSA control (Figure S1). This concentration was used for all subsequent experiments unless stated otherwise. An experiment was then conducted to determine the duration and peak for elevated TGF-β1 following LTBP-2 stimulation. After an addition of exogenous LTBP-2, the TGF-β1 content of the medium was measured at intervals over 24 h (Figure 3A). A significant increase in TGF-β1 above controls was first observed after 9 h. At the 12 h time point, the LTBP-2-treated cells showed around a twofold increase in TGF-β1 above controls, which rose to threefold at 15 and 24 h. Since TGF-β1 secretion was significantly higher by control cells at 24 h compared to 15 h, the TGF-β1 levels appeared to peak at around 15 h. No significant differences were found in cell numbers in the LTBP-2-treated cultures and untreated controls, although the cell count in all cultures had doubled after 24 h of incubation (Figure 3B). Therefore, the LTBP-2-induced stimulation of TGF-β1 secretion was not due to a greater increase in cell number in the LTBP-2-treated cultures.

### 2.3. A Short Exposure of Cells to Exogenous LTBP-2 Is Sufficient to Stimulate TGF-β Upregulation

**Figure 4:**
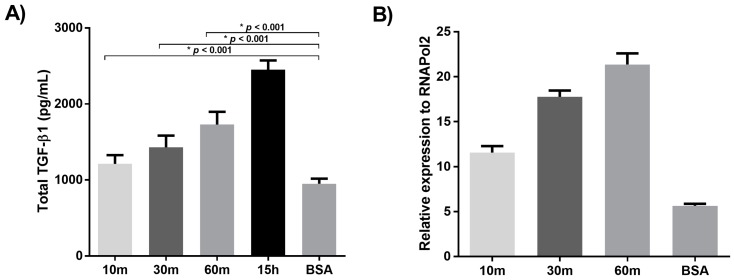
A short incubation of MSU-1.1 cells with LTBP-2 is sufficient to upregulate TGF-β1 expression and secretion. (**A**) Quantitation of TGF-β1 in the medium. MSU-1.1 cells were grown to 24 h post-confluence, then incubated in serum-free medium with LTBP-2 (10 µg/mL) for various time periods (10–60 min). After each incubation period, the medium was discarded and replaced with fresh serum-free medium lacking LTBP-2. The conditioned medium was collected at 15 h and analysed for TGF-β1 content (see Materials and Methods). The results were compared to cells incubated for 15 h with LTBP-2 or a BSA control. A significant increase in TGF-β1 was detected with as little as 10 min of exposure to LTBP-2. The mean values of three independent experiments are shown (±SD); (**B**) Quantitation of TGF-β1 mRNA. At the end of each 15 h incubation above, total RNA was harvested from the cells, then reverse transcribed into cDNA. Quantitative PCR was performed to determine cellular TGF-β1 mRNA levels as described in materials and methods. Values were expressed relative to the *RNAP2* housekeeping gene. The control consisted of cells exposed to BSA instead of LTBP-2 for 15 h. A significant increase in TGF-β1 mRNA was detected after 15 h with as little as 10 min of LTBP-2 exposure.

To determine if the LTBP-2-induced elevation of TGF-β1 was a direct effect of LTBP-2 on the cells, we tested if a short incubation with LTBP-2 was sufficient to raise TGF-β1 levels in the medium. MSU-1.1 cells were incubated with exogenous LTBP-2 for time periods of 10 to 60 min before the LTBP-2 was removed by changing the medium. After 15 h, the medium was analysed for TGF-β1 content (Figure 4A). Interestingly exposure of cells to LTBP-2 for as little as 10 min caused a small but significant increase in TGF-β1 in the medium after 15 h, suggesting that the effect was mediated by the direct interaction of LTBP-2 with the cell surface. Exposure of the cells to LTBP-2 for 30 min and 60 min further elevated TGF-β levels in the medium, with a 60 min exposure to LTBP-2 causing around 50% of the increase resulting from incubation with LTBP-2 for 15 h (Figure 4A).

To determine if the LTBP-2 stimulated increase in TGF-β1 secretion correlated with an increased cellular expression of TGF-β1, TGF-β1 mRNA levels in the fibroblasts were measured at 15 h after short exposure times to LTBP-2. The cell layer was harvested after 15 h, and total RNA was extracted and reverse transcribed for qPCR (Figure 4B). Exposure of the cells to LTBP-2 for as little as 10 min resulted in a significant elevation of TGF-β1 mRNA above the BSA controls. Incubation with LTBP-2 for 30 and 60 min caused additional increases in TGF-β1 mRNA levels, with the 60 min exposure causing an approximately fourfold increase in TGF-β1 above control cells. These results indicate that a short exposure of MSU-1.1 cells to exogenous LTBP-2 is sufficient to stimulate TGF-β1 expression and secretion, strongly suggesting that the process is directly mediated by the interaction of LTBP-2 with (a) cell surface receptor(s) to trigger intracellular signalling pathways.

### 2.4. The TGF-β1 Stimulating Activity Maps to a Central Region of LTBP-2 Consisting of an Eight-Cys Motif Flanked by Pairs of EGF-Like Repeats

**Figure 5:**
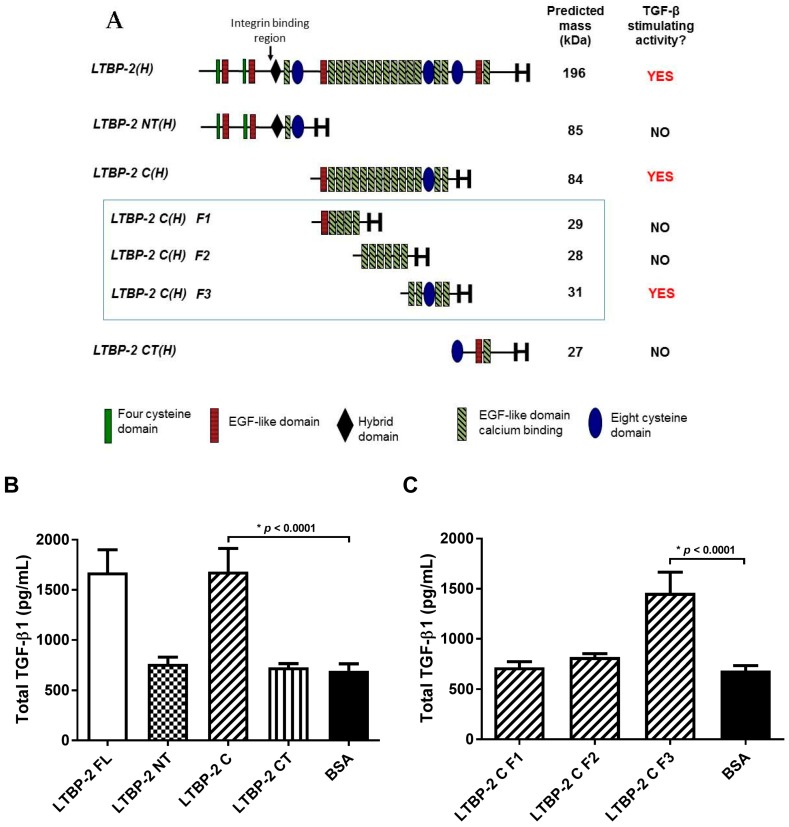
A central region of LTBP-2 consisting of an 8-cys motif flanked by pairs of epidermal growth factor (EGF)-like repeats (fragment LTBP-2C F3) contains the TGF-β1 stimulatory activity. (**A**) Schematic diagram of recombinant LTBP-2 fragments. Protein fragments produced specifically for this study (LTBP-2C(H) F1, F2, and F3) are highlighted within the blue box; (**B**) MSU-1.1 cells were grown to 24 h post-confluence, then incubated for 16 h with full-length LTBP-2 (50 nM), molar equivalents of each of three fragments spanning LTBP-2 (LTBP-2NT, LTBP-2C, LTBP-2CT) or BSA control. The mean values of two independent experiments are shown (±SD); (**C**) Fresh cells were subsequently incubated with each of three sub-fragments F1, F2, and F3 spanning central fragment LTBP-2C(H). In both (**A**) and (**B**), the conditioned medium was analysed for TGF-β1 content (see Materials and Methods). The mean values of two independent experiments are shown (±SD).

To identify the region(s) of LTBP-2 responsible for TGF-β1 elevation, a series of LTBP-2 fragments were tested in place of full-length LTBP-2 in exogenous addition experiments. Initially, three recombinant fragments, designated LTBP-2NT(H), LTBP-2C(H), and LTBP-2CT(H) [14], spanning the LTBP-2 molecule were used (Figure 5A). After 16 h, the TGF-β1 content of medium from cells treated with fragments LTBP-2NT(H) or LTBP-2CT(H) was similar to BSA-treated controls. However, the central fragment LTBP-2C(H) caused a significant increase in TGF-β1, similar to full-length LTBP-2 (Figure 5B), indicating that the relevant bioactive region of LTBP-2 was in the centre of the molecule and away from known integrin binding activity in the N-terminal region [28].

Subsequently, three sub-fragments F1, F2, and F3, spanning fragment LTBP-2C(H), were tested in a TGF-β1 bioassay. Only fragment F3, consisting of an 8-Cys motif flanked by two pairs of EGF-like repeats, caused a significant increase in TGF-β1 secretion above control cells, indicating that it contained the TGF-β1 stimulating activity (Figure 5C). This region has no known cellular or molecular interactions, although it is adjacent to a region containing heparin and fibroblast growth factor-2 (FGF-2) binding activities (Fragment F2) [29,30].

### 2.5. Induction of Akt and p38 MAPK Phosphorylation by LTBP-2

**Figure 6:**
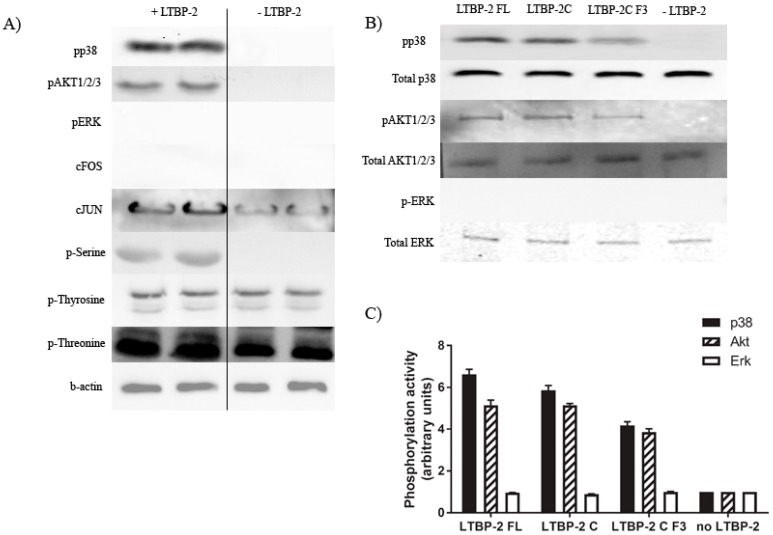
Exogenous LTBP-2 stimulates phosphorylation of AKT and p38 mitogen-activated protein kinase (MAPK) in human fibroblasts. (**A**) MSU-1.1 cells (1 × 10^5^ cells/well) were treated with or without LTBP-2 (10 µg/mL) for 30 min. Total cell lysates were immunoblotted for phosphorylation of candidate signalling molecules, including phospho-serine, phospho-tyrosine, phospho-threonine, phospho-p38 MAPK, phospho-Akt1/2/3, phospho-ERK, c-FOS, c-JUN, and for β-actin internal control as described in materials and methods. Note that there was major phosphorylation of p38 MAPK and AKT1/2/3, but no stimulation of ERK or cFOS; (**B**) Cells were treated for 30 min with full-length LTBP-2 or with molar equivalents of fragments containing TGF-β1 stimulating activity, LTBP-2C(H), or LTBP-2C(H) F3. Cell lysates were immunoblotted for total and phosphorylated p38 MAPK, Akt1/2/3, and ERK; (**C**) The ratio of phospho-protein to total protein for each signal molecule from each treatment is expressed relative to the average value from no LTBP-2 control cells (given an arbitrary value of 1.0). Similar results were observed in replicate experiments.

To determine the signalling pathways involved in the stimulation of TGF-β1 expression, we examined the effects of LTBP-2 on the phosphorylation of serine, tyrosine, and threonine generally and on targeted individual signal pathways, namely cJUN, cFOS, AKT1/2/3, ERK, and p38 MAPK. These signalling molecules were selected to cover the main pathways commonly used by integrins, growth factors, and cytokine receptors. Western blotting (Figure 6A) showed that the treatment of MSU-1.1 cells with LTBP-2 (1 µg/mL) for 30 min increased the phosphorylation of serine, threonine, cJUN, Akt1/2/3, and p38 MAPK. However, we observed no increase in tyrosine, cFOS, and ERK phosphorylation. Thus, LTBP-2 seems to specifically activate signalling molecules containing phospho-serine and threonine, and pathways involving AKT and p38 MAPK. The experiment was repeated using bioactive fragment LTBP-2C and sub-fragment LTBP-2CF3 in place of full-length LTBP-2. Phosphorylation was quantitated relative to the no LTBP-2 control after normalisation to the total signal molecule present. The bioactive fragments activated Akt and p38 MAPK signalling but not ERK, similar to full-length LTBP-2 (Figure 6B,C), leading to the hypothesis that LTBP-2 may stimulate TGF-β1 expression by activating these two signalling pathways.

### 2.6. LTBP-2 Stimulates the Expression of TGF-β1 via Akt and p38 MAPK Signalling Pathways

**Figure 7:**
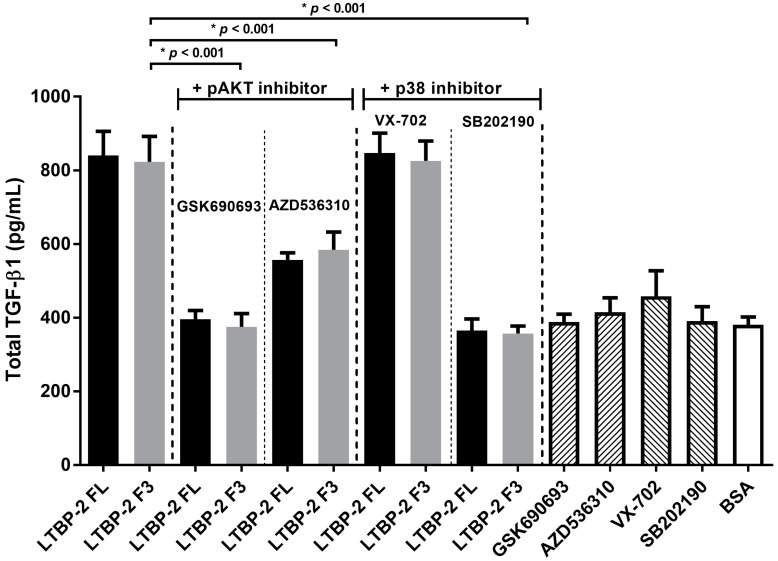
LTBP-2 stimulation of TGF-β upregulation involves Akt and p38 MAPK signalling pathways. MSU-1.1 cells were grown to 24 h post-confluence, then incubated in serum-free medium with or without inhibitor (10 µM) of Akt (GSK690693 or AZD536310) or p38 MAPK (VX-702 or SB202190) for 2 h, followed by an addition of 50 nM of LTBP-2 or bioactive fragment LTBP-2C F3. Incubation was continued for a further 15 h, and the conditioned medium was analysed for TGF-β1 content (see Materials and Methods). Controls involved incubations with individual inhibitors or BSA in the absence of LTBP-2. The mean values ± SD from duplicate experiments are shown.

Specific inhibitors were used to determine if the LTBP-2-induced phosphorylation of AKT1/2/3 and/or p38 MAPK caused an upregulation of TGF-β1. MSU-1.1 cells treated with or without LTBP-2 were cultured in the presence of an Akt phosphorylation inhibitor, GSK690693 or AZD5363, or a p38 MAPK phosphorylation inhibitor, SB202190 or VX-702, and TGF-β1 in the medium was measured by ELISA after 16 h (Figure 7). The LTBP-2-induced upregulation of TGF-β was inhibited by AKT phosphorylation inhibitors GSK690693 and AZD536310, and inhibited by p38 MAPK inhibitor (SB202190) but not inhibitor VX-702. SB202190 blocks phosphorylation of both the α and β forms of p38 MAPK, whereas VX-702 blocks only the α isoform [31]. Thus, it appears that the LTBP-2-induced upregulation of TGF-β1 involves AKT1/2/3 and p38β MAPK.

### 2.7. Blocking of Integrin αVβ3 Receptors Partially Attenuates TGF-β1 Production by LTBP-2

**Figure 8:**
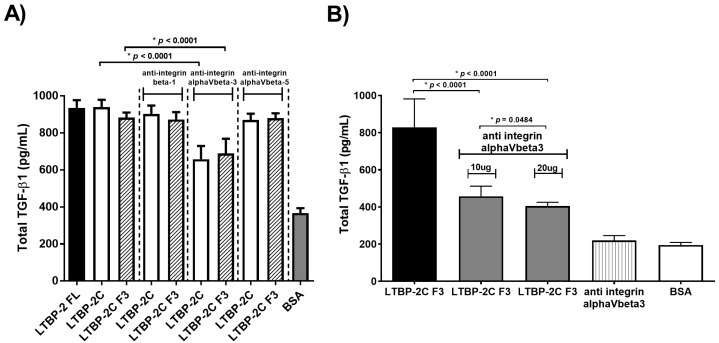
Blocking of integrin αVβ3 receptors partially attenuates TGF-β1 production induced by LTBP-2. (**A**) Effects of blocking antibodies to integrins on LTBP-2 stimulated TGF-β1 expression. MSU-1.1 cells were pre-treated with blocking antibody for integrins β1 (10 µg/mL), αVβ3 (10 µg/mL), and αVβ5 (10 µg/mL) for 2 h. Incubation was continued for 15 h with the addition of a bioactive LTBP-2 fragment (LTBP-2C(H) or LTBP-2C(H) F3) 50 nM final concentration), and the conditioned medium was analysed for TGF-β1 content (see Materials and Methods). Control incubations included LTBP-2 or a bioactive fragment without antibody and BSA in the absence of LTBP-2. The mean values of two independent experiments are shown (±SD); (**B**) Increasing the concentration of anti-integrin αVβ3 antibody caused little further attenuation of LTBP-2-induced TGF-β1 upregulation. The above experiment was repeated including two concentrations of anti-integrin αVβ3 (10 µg/mL and 20 µg/mL) during a 2 h pre-incubation and for a further 15 h following an addition of a LTBP-2 C F3 fragment (5 nM final concentration). The conditioned medium was then analysed for TGF-β1 content (see Materials and Methods). Controls involved incubation of cells with anti-integrin antibody only or BSA. Mean values ±SD from triplicate wells are shown. Doubling of the anti αvβ3 antibody concentration caused only a very slight additional reduction in TGF-β stimulation, suggesting that the blocking effect was close to saturation. The mean values of two independent experiments are shown (±SD).

To determine if an integrin was involved in the upregulation of TGF-β, a range of established blocking antibodies was tested in the LTBP-2 stimulation assay (Figure 8A). Antibodies to the β1 and αVβ5 integrins had no effect on LTBP-2 upregulation of TGF-β1, indicating that the large family of β1 integrins and αVβ5 integrin were not evidently involved in the process. Intriguingly, the anti-αVβ3 integrin antibody (10 µg/mL) showed partial inhibition of the TGF-β1’s upregulation. In a subsequent experiment, doubling of the anti-αVβ3 antibody concentration and reducing the concentration of LTBP-2F3 10-fold showed only a very minor further attenuation, suggesting that the inhibitory effect of the antibody was close to saturation (Figure 8B). Even under these conditions, LTBP-2 still elevated TGF-β levels to double the amount in control cultures. This suggested that αVβ3 integrin alone was not responsible for the signal transmission and that another primary receptor was involved, perhaps in conjunction with αVβ3, to mediate TGF-β1 upregulation by LTBP-2.

## 3. Discussion

To our knowledge, the current study is the first to investigate the mechanism of TGF-β regulation by LTBP-2. Our initial hypothesis was that excess LTBP-2 would compete with LTBP-1 for binding and release the TGF-β1 complex from its storage sites in the matrix. This was investigated by an incubation of exogenous LTBP-2 with MSU-1.1 cells that had elaborated an extensive network of fibrillin-microfibrils. A protein causing the release of TGF-β complexes from matrix storage was previously documented by Chaudhry et al., who found that a fragment of fibrillin-1 caused a significant increase in TGF-β1 bioavailability by inhibiting the binding of LTBP-1 to fibrillin microfibrils [32]. Our experiments showed a significant increase in latent TGF-β1 in the culture medium, but there was no significant decrease in TGF-β1 in the cell layer. Moreover, repeating the experiment with cell cultures containing a very limited matrix gave a similar result, suggesting that an alternative mechanism was responsible for the elevation of TGF-β1. An addition of cycloheximide blocked the increase in TGF-β1, confirming that new protein synthesis was required. Exogenous LTBP-2 was subsequently shown to cause a fourfold increase in TGF-β1 mRNA in the fibroblasts, confirming that the increase in TGF-β1 in the medium was due to an increased expression and secretion of new TGF-β1 rather than a release of matrix-stored growth factor. Following an addition of exogenous LTBP-2, the increase in TGF-β1 could only be detected after 9 h, and TGF-β1 levels peaked at around 15 h. This long time frame is consistent with LTBP-2 stimulating *TGFB1* gene expression and the secretion of new TGF-β1 into the medium. This result contrasts with the Chaudhry et al. study, where a fibrillin fragment displaced TGF-β1 from matrix storage after as little as 10 min [32].

Exposure of MSU-1.1 cells to exogenous LTBP-2 for only 10 min resulted in increased TGF-β1 expression and secretion, strongly suggesting that LTBP-2 was directly interacting with the cells via (a) receptor(s), or possibly an integrin, to elicit the response rather than displacing other bioactive molecules, such as growth factors, from matrix storage. Since LTBP-2 has known integrin-binding activity [28], it was important to determine if the TGF-β1 upregulation involved known LTBP-2-cell interactions. The TGF-β1 stimulating bioactivity was principally confined to a short region in the centre of LTBP-2, consisting of an 8-Cys motif flanked on each side by a pair of EGF-like repeats (fragment F3) (see Figure 5A). This was clearly distinct from the integrin binding site with affinity for the integrins α3β1 and α6β1, situated towards the N-terminus [28]. To determine if an integrin was, or integrins were, involved in LTBP-2-induced TGF-β1 upregulation, we included inhibitory antibodies to block specific integrin activity throughout the bioassay. Antibodies to the integrins αVβ5 and β1 showed no inhibitory effect. An anti-αVβ3 integrin antibody partially inhibited LTBP-2 upregulation of TGF-β1, which was surprising since no RGD sequence is present in the bioactive LTBP-2 sequence. However, only partial blocking of the TGF-β1 upregulation occurred even with a large excess of anti-αVβ3 antibody. This suggested that, while the LTBP-2 bioactivity may indirectly involve active αVβ3 integrin, another main receptor was, or other main receptors were, responsible for primary signal transmission. Integrins can stimulate signalling pathways independently, but more often they act synergistically with other receptors to elicit a full signalling response [33,34]. Specific integrins can interact with receptors for vascular endothelial growth factor, TGF-β, insulin, EGF, and platelet-derived growth factor-β, or with cell surface heparan sulphate proteoglycans (HSPGs) [35]. The integrin αVβ3 modulates platelet-derived growth factor-β, a potent stimulator of cell motility, and this interaction appears to have an antagonistic effect on the reactive oxygen species production at focal adhesion sites [36]. In addition, the α5β1 integrin interacts with the cell surface HSPG, syndecan-4, to regulate matrix structure and cell adhesion during development and in most adult tissues [37]. It is possible that the bioactive region of LTBP-2 (fragment F3) binds to an unidentified main receptor or co-receptor in complex with the αVβ3 integrin. The bound anti-αVβ3 antibody might partially obscure the binding of LTBP-2 to its main receptor without LTBP-2 being a direct ligand for the αVβ3 integrin.

We also investigated if any major intracellular signalling pathways were activated by LTBP-2 resulting in TGF-β upregulation. LTBP-2 increased the phosphorylation of specific signalling proteins, namely p38 MAPK and Akt1/2/3 (but not ERK). The use of specific inhibitors indicated that both molecules were important LTBP-2-induced upregulation. The p38 MAPK family consists of four members, p38α p38β, p38γ, and p38δ [38]. P38 MAPK is strongly activated by such stimuli as environmental stress, inflammatory cytokines, and growth factors [38], and is involved in pathological conditions including inflammation, rheumatoid arthritis, cancer, cardiac hypertrophy, neurodegenerative disorders, and fibrosis [39]. Only p38β inhibitor (SB2021900) inhibited LTBP-2-induced TGF-β1 upregulation, indicating that this isoform is involved in the cascade. A main feature of p38β MAPK activation is sustaining cell survival [40]. For example, P38β MAPK induces a potent anti-apoptotic factor in the synovial membrane of rheumatoid arthritis patients [41]. Activation of p38β MAPK is important in the anti-inflammatory, anti-proliferative, and anti-apoptotic effects of carbon monoxide in various models [42].

Akt1/2/3 is a family of serine/threonine-specific protein kinases that plays key roles in the regulation of cell survival, cell cycle, and growth [43]. Akt signalling components are frequently altered in human cancers and in fibrosis [44,45]. For instance, Li et al., demonstrated that Akt pathway activation promoted collagen production and scar formation in acute muscle contusion [46]. Akt pathway activation can also promote lung fibroblast proliferation and pulmonary fibrosis by enhancing macrophage survival [47]. Interestingly, inhibitor GSK690693 more markedly blocked the upregulation of TGF-β1 by Akt phosphorylation compared to inhibitor AZD536310. GSK690693 (IC 50 = 2 nM) is a more potent Akt1 inhibitor than AZD5363 (IC 50 of 3 nM), suggesting that Akt1 may be the main Akt isoform involved here. Akt1 is associated with altered cell invasion and migration, and is a frequently activated protein kinase in cancers [48] where it enhances the invasion capability of cancer cells. Akt is considered a potential therapeutic target for several cancer types [49]. Akt1 is also linked to cardiac fibrosis by promoting inflammatory response in early stages of hypertensive heart disease [50].

From our study, both Akt and p38β MAPK appear to be essential for TGF-β1 upregulation by LTBP-2, which is consistent with other studies linking these pathways to TGF-β1 regulation. Blockade of the Ras/PI3K/Akt pathways reduced the expression of TGF-β as an angiogenic factor in mouse osteosarcoma [51] and suppressed TGF-β1 production by macrophages treated with phophatidylserine-liposomes [52]. Interestingly, Xiao et al., showed that apoptotic cells upregulate TGF-β mRNA transcription via activation of the p38 MAPK pathway and TGF-β1 mRNA translation via activation of the Akt pathway [53]. If LTBP-2 used similar mechanisms to upregulate TGF-β1 expression, it would explain why extensive inhibition was observed by the blocking of either p38 MAPK or Akt. It should be noted that we have not established that the LTBP-2-induced p38 MAPK and Akt phosphorylation leads to a direct stimulation of TGF-β1 expression. Given the observed delay of several hours in TGF-β1 upregulation by LTBP-2, it is possible that expression of (an) intermediate protein(s) is stimulated that in turn upregulates the expression of TGF-β1. It is noteworthy that a very recent paper has shown that LTBP-2 knockdown inhibits invasion and tumorigenesis in thyroid cancer cells and this involves the PI3K/Akt signalling pathway [54]. Further elucidation of the above pathways and their control mechanisms will enhance our knowledge of fibrosis and other diseases associated with TGF-β dysregulation.

**Figure 9:**
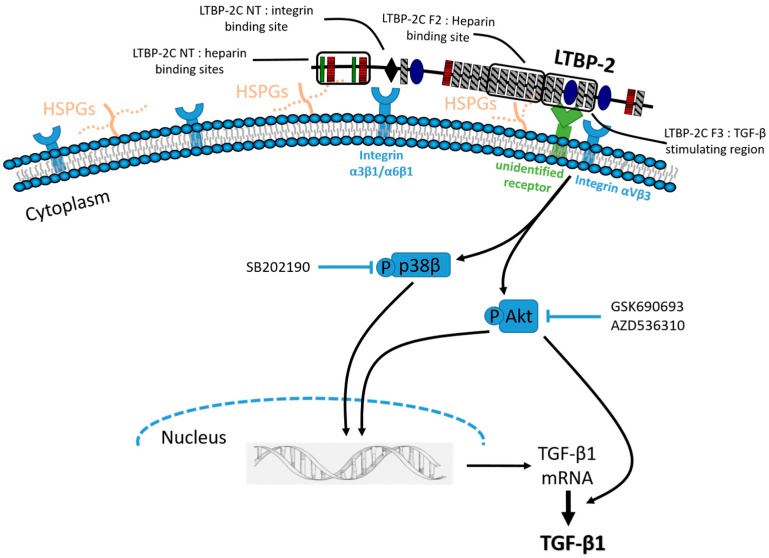
Schematic model of possible signalling events involved in the upregulation of TGF-β1 expression by LTBP-2. LTBP-2 binds to an unidentified receptor (perhaps complexed with αVβ3 integrin and enhanced by binding to cell surface heparan sulphate proteoglycans (HSPGs)) and induces the phosphorylation of Akt and p38β MAPK pathways. The inhibition of either pathway inhibits TGF-β1 expression and secretion. The precise mechanisms remain to be elucidated, but in other systems blocking p38 MAPK has been shown to inhibit *TGFB1* gene transcription and blocking Akt pathways can inhibit TGF-β1 transcription and/or translation. Arrows indicate positive (stimulatory) effects. The inhibitors used to specifically block respective signalling molecules are also depicted.

It is interesting that full-length LTBP-2 and the larger fragment LTBP-2C caused a stronger activation and phosphorylation of the Akt and p38 MAPK signalling pathways than the smaller fragment LTBP-2 F3. This suggests that a region adjacent to F3 may enhance its signalling properties, perhaps by positively influencing the binding of LTBP-2 to the cell surface. A candidate molecule for this is a cell surface HSPG. Various HSPGs are found at the cell surface and/or the extracellular matrix and play important roles as cell receptors and co-factors, and they can act in conjunction with integrins to modulate cell behaviour and signalling outcomes, often through growth factor receptor signalling pathways [33,35,55]. Interestingly, our recent studies revealed that LTBP-2 binds strongly to HSPGs, including cell-signalling, transmembrane syndecan-4, with multiple binding sites within the N-terminal region of LTBP-2 and one site (in fragment LTBP-2F2) adjacent to the TGF-β stimulating region (fragment LTBP-2F3) [30]. Binding of LTBP-2 to HSPG via this adjacent site may explain the stronger signalling invoked by full-length LTBP-2 and fragment LTBP-2C than by LTBP-2F3, which lacks HSPG-binding activity. The concepts are illustrated schematically in Figure 9. Further research is needed to elucidate the full nature of LTBP-2 interaction(s) with the cell surface required for TGF-β1 upregulation.

The identification of the LTBP-2 receptor involved in TGF-β1 upregulation may prove difficult. For instance, the Akt signaling cascade can be activated by a variety of integrins, cytokine receptors, immune cell receptors, receptor tyrosine kinases, and G-protein-coupled receptors [56]. At face value, the results indicate that receptor(s) not signalling primarily via ARK or p38 are unlikely to be prime candidates for the unidentified LTBP-2 receptor. For example, growth factors EGF, FGF, and Platelet-Derived Growth Factor primarily use ERK pathways [57,58], and the TGF-β/BMP family uses the SMAD signalling cascade [59]. However, such growth factors can also use non-canonical pathways; EGF can stimulate Akt via mTORC2 [60], and TGF-β1 can use several signalling pathways involving p38, Akt, or ERK [59,61]. Therefore, caution is required when eliminating possible candidates for the unidentified LTBP-2 receptor.

Since TGF-β1 plays a key role in the proliferative phase of wound healing and in fibrosis [62], it is possible that the TGF-β1 upregulation by LTBP-2 in fibroblasts plays some role in fibrotic disease processes and in genetic disorders which have fibrotic components, such as WMS [20]. We have recently shown that LTBP-2 is highly expressed in fibrotic skin conditions, keloids, and hypertrophic scars [29,63]. Interestingly, LTBP-2 co-localised in these tissues with another growth factor, FGF-2. LTBP-2 is a potent inhibitor of FGF-2 activity via a single high-affinity binding site in the fragment LTBP-2CF2 [29,63]. This region corresponds to a *LTBP2* gene mutation linked to a case of WMS [20]. It is possible that elevated LTBP-2 might influence FGF-2 bioactivity during wound repair and healing processes and in fibrosis. Clearly, more research is required to fully establish the influence of LTBP-2 on TGF-β1 and FGF-2 bioactivities in fibrosis and fibrotic diseases.

## 4. Materials and Methods

### 4.1. Reagents

LTBP-2 and fragments were produced using the pCEP4 expression vector in 293EBNA cells as described previously [14,29,30]. Gels showing the size and purity of each fragment have been published elsewhere [29]. Antibodies against p-Akt1/2/3 (Ser473), Akt1/2/3 (H-136), p-p38 (D-8), p38 (H-147), p-ERK (E-4), ERK (MK1), and β-actin (C4) were purchased from Santa Cruz Biotechnology (Santa Cruz, CA, USA); Dylight-800 labelled anti-mouse and anti-rabbit IgGs were from Thermo Scientific, Waltham, MA, USA. Rabbit anti-[human LTBP-2 peptide] antibody 3504 has been described previously [14]. Signalling inhibitors SB202190 and VX-702 for p38 MAPK and GSK690693 and AZD5363 for Akt were from Selleckchem, (Houston, TX, USA). Blocking antibodies for β1 integrin (P4C10) and αVβ3 integrin (CD51/61) were from Millipore (Temecula, CA, USA); anti-αVβ5 integrin (P1F6) was from Abcam (Cambridge, UK).

### 4.2. Cell Culture and Treatment

His6-tagged full-length r-LTBP-2 and contiguous fragments were produced in 293-EBNA cells and purified from serum-free culture medium by using Ni-Sepharose as described previously [14,29,30]. Purified recombinant proteins were dialyzed into tris-buffered saline (TBS)–0.5 M NaCl and analysed by gel electrophoresis and immunoblotting to confirm size and purity.

MSU-1.1 fibroblasts were grown for up to 3 weeks post-confluence in 6-well plates in Dulbecco’s Modified Eagle’s Medium plus 10% FCS at 37 °C. The cells were incubated for various times (3–24 h) in serum-free medium with r-LTBP-2 (final concentration 2.5–12.5 µg/mL). For control wells, BSA was added instead of LTBP-2. In some instances, protein synthesis was inhibited with cycloheximide (10 µg/mL) (Sigma, St. Louis, MO, USA). In the signalling studies, cells were pre-incubated for 2 h with Akt or p38 MAPK inhibitors (10 mM) prior to an addition of LTBP-2. The cell layer was resuspended in 1 mL PBS, and treated with 1 M HCl (200 µL) to activate latent TGF-β. After 10 min, the suspension was neutralised with 1.2 M NaOH/0.5 M HEPES. After centrifugation for 5 min at 350 *g*, the supernatant was analysed for TGF-β1 content by ELISA.

In the initial integrin study, the cells were pre-incubated for 2 h with blocking antibodies to integrin β1, αVβ3, or αVβ5 (10 µg/mL) prior to an addition of LTBP-2, fragment LTBP-2C, or fragment LTBP-2 F3 (50 nM). The cells were then incubated for 15 h in the presence of antibody. For control wells, BSA (10 µg/mL) was added instead of LTBP-2. The TGF-β1 content of the medium was determined by ELISA. In the subsequent experiment cells were incubated with fragment LTBP-2 F3 at 5 nM and anti-αVβ3 at 10 µg/mL or 20 µg/mL.

### 4.3. Detection and Quantitation of TGF-β

The TGF-β1 content of conditioned medium was determined using the DuoSet ELISA for human TGF-β1 (R&D Systems, Minneapolis, MN, USA), following the manufacturer’s instructions. Briefly, wells were coated with mouse anti-TGF-β1 (200 ng in PBS) and incubated for 18 h. The wells were washed with 0.05% Tween 20 in PBS, blocked with 5% Tween 20 in PBS for 1 h, and incubated with conditioned medium (100 µL) for 2 h. Control wells were incubated with fresh medium. For standard curves, wells were incubated with TGF-β1 (100 µL) (31.3 to 2000 pg/mL) in 1.4% BSA in PBS. With washing between steps, wells were incubated with 100 µL of biotinylated chicken anti-human TGF-β1, (300 ng/mL) for 2 h, then streptavidin-horseradish peroxidase (100 µL) for 20 min, followed by 100 µL Substrate Solution (R&D Systems) for 20 min, in reduced lighting. The reaction was stopped with 50 µL 1 M H_2_SO_4_, and absorbance (450 nm) was measured using a Titertek Multiscan microplate reader (Flow Laboratories).

### 4.4. Quantitative PCR

Total RNA was extracted from cells using trizol (Invitrogen, California, CA, USA), following the manufacturer’s instructions. RNA samples were treated with DNase 1 (RQ1 DNase, Promega, Fitchburg, WI, USA) and recovered using the RNEasy Mini Kit (Qiagen, Düsseldorf, Germany). Total RNA (1 µg) was reverse transcribed using Superscript III (Invitrogen, Carlsbad, CA, USA). The cDNA was PCR-amplified using a SYBR-Green kit (Qiagen). Primer sequences (forward and reverse respectively) were as follows: TGF-β 5′-CTCCGAGAAGCGGTACCTGAAC-3′, and 5′-CACTTGCAGTGTGTTATCCCT-3′; LTBP-2 5′-GGGCACCGCACCACCTACACG-3′ and 5′-TCATCACACTCATTCACATCTACG-3′; RNAPolII 5′-AGGGGCTAACAATGGACACC-3′ and 5′-CCGAAGATAAGGGGAACTACT-3′. The housekeeping gene *RNAP2* was used as the internal standard. PCR was performed with a hot start at 95 °C for 10 min, followed by 40 cycles of denaturation at 95 °C for 20 s, annealing at 60 °C for 20 s, and extension at 72 °C for 45 s.

### 4.5. Measurement of Signal Phosphorylation

MSU-1.1 cells (4 × 10^5^ cells/well) were treated with LTBP-2 (50 nM, 10 µg/mL) for 30 min. Cells were then lysed with 50 mM Tris buffer (pH 6.8) containing 0.5% SDS and 2 mM EDTA plus phosphatase and protease inhibitors (#04906837001 and #11836153001, Roche Diagnostics, Basel, Switzerland). The protein content of cell extracts was measured using a BCA Kit (Thermo Fisher Scientific, Waltham, MA, USA). Extracted protein (50 µg) was analysed on 12% gels and transferred to nitrocellulose (Pall Corporation, Port Washington, NY, USA). The membrane was blocked with 10% milk in tris-buffered saline (TBS) for at least 1 h and then incubated with a primary antibody to a phosphorylated or total signalling molecule (AKT, P-38 MAPK, or ERK) in 2% milk at 4 °C overnight. The membrane was washed in TBS then incubated with secondary anti-rabbit IgG or anti-mouse IgG antibodies conjugated with IR800 fluorescence dye, (1:5000 dilution), in 3% milk for 1 h. Membranes were imaged with the LI-COR Odyssey Infrared Imaging System. Bands were quantitated using ImageJ 1.48 software (NIH, Bethesda, MD, USA) and normalised to the internal β-actin signal or total protein. For comparison of the phospho- and total signals, the ratio of normalised phospho-protein signal to the total protein signal was expressed in arbitrary units relative to the average value from cells incubated without LTBP-2 treatment (equalling 1).

### 4.6. Statistical Analysis

The data from in vitro TGF-β stimulation assays, cell count assays, Western blot quantitation, and qPCR were statistically analysed using a paired Student’s *t*-test with GraphPad Prism 7.02 software. A *p* value ≤ 0.05 was regarded as statistically significant.

## Supplementary Materials

Supplementary materials can be found at www.mdpi.com/1422-0067/18/10/2114/s1.

## Abbreviations

LTBP-2	Latent Transforming Growth Factor β-1 Binding Protein 2
TGF-β	Transforming Growth Factor β
EGF	Epidermal Growth Factor
FGF	Fibroblast Growth Factor
MAPK	Mitogen-Activated Protein Kinase
TBS	tris-Buffered Saline
PBS	Phosphate-buffered Saline
BSA	Bovine Serum Albumin
ELISA	Enzyme-Linked Immunosorbent Assay
HSPG	Heparan Sulphate Proteoglycan
WMS	Weill–Marchesani Syndrome
